# β-Cyclodextrin conjugated bifunctional isocyanate linker polymer for enhanced removal of 2,4-dinitrophenol from environmental waters

**DOI:** 10.1098/rsos.180942

**Published:** 2018-08-29

**Authors:** J. M. Anne, Y. H. Boon, B. Saad, M. Miskam, M. M. Yusoff, M. S. Shahriman, N. N. M. Zain, V. Lim, M. Raoov

**Affiliations:** 1 School of Chemical Sciences, Universiti Sains Malaysia, 11800, Pulau Pinang, Malaysia; 2 Integrative Medicine Cluster, Advanced Medical and Dental Institute, Universiti Sains Malaysia, 13200 Bertam, Pulau Pinang, Malaysia; 3 Department of Fundamental and Applied Sciences, Faculty of Science and Information Technology, Universiti Teknologi Petronas, 32610 Seri Iskandar, Perak Darul Ridzuan, Malaysia; 4 Department of Chemistry, Faculty of Science, Universiti Malaya, 50603 Kuala Lumpur, Malaysia; 5 Universiti Malaya Centre for Ionic Liquids (UMCIL), Department of Chemistry, Faculty of Science, Universiti Malaya, 50603 Kuala Lumpur, Malaysia

**Keywords:** cross-linked polymer, aromatic linker, aliphatic linker, adsorption, toxic contaminants

## Abstract

In this work, we reported the synthesis, characterization and adsorption study of two β-cyclodextrin (βCD) cross-linked polymers using aromatic linker 2,4-toluene diisocyanate (2,4-TDI) and aliphatic linker 1,6-hexamethylene diisocyanate (1,6-HDI) to form insoluble βCD-TDI and βCD-HDI. The adsorption of 2,4-dinitrophenol (DNP) on both polymers as an adsorbent was studied in batch adsorption experiments. Both polymers were well characterized using various tools that include Fourier transform infrared spectroscopy, thermogravimetric analysis, Brunauer–Emmett–Teller analysis and scanning electron microscopy, and the results obtained were compared with the native βCD. The adsorption isotherm of 2,4-DNP onto polymers was studied. It showed that the Freundlich isotherm is a better fit for βCD-TDI, while the Langmuir isotherm is a better fit for βCD-HMDI. The pseudo-second-order kinetic model represented the adsorption process for both of the polymers. The thermodynamic study showed that βCD-TDI polymer was more favourable towards 2,4-DNP when compared with βCD-HDI polymer. Under optimized conditions, both βCD polymers were successfully applied on various environmental water samples for the removal of 2,4-DNP. βCD-TDI polymer showed enhanced sorption capacity and higher removal efficiency (greater than 80%) than βCD-HDI (greater than 70%) towards 2,4-DNP. The mechanism involved was discussed, and the effects of cross-linkers on βCD open up new perspectives for the removal of toxic contaminants from a body of water.

## 1. Introduction

To date, a lot of new adsorbents based on natural and polymeric materials have been developed in order to remove organic and inorganic pollutants from water distribution networks. Numerous approaches have been studied for the development of cheaper and more effective adsorbents, for example activated carbon, which is the most widespread adsorbent being used in water treatment. However, they have several deficiencies, including slow pollutant uptake and poor removal of hydrophilic pollutants. This phenomenon has led to the invention of cross-linking polymers which have gained great interest in various applications such as synthesis, extraction, tissue engineering, drug delivery, and in other pharmaceutical and biomedical applications. Furthermore, the growing interest in supramolecular chemistry has allowed us to prepare polymers of β-cyclodextrin (βCD), which is inexpensive with high regeneration values [1,2], sustainably producing macrocycles of glucose, which is of interest for removing pollutants from water by means of adsorption. As the parental CDs are soluble in water, polymerization works with bifunctional linkers; therefore, it is necessary to make them insoluble in water. The CD molecules are natural macrocyclic polymers, formed by the action of an enzyme on starch. The three smallest CDs, αCD, βCD and γCD, which consist of six, seven and eight α-1,4 linked d(C)-glucopyranose units with numerous available hydroxyl groups, are active sites for forming different types of linkages and derivatives [3–5].

CDs are truncated cone-shaped polymers rather than cylindrical due to the conformation of glucopyranose units. The most characteristic feature of CD is the ability to form inclusion compounds with various molecules, especially aromatics. The interior cavity of the molecule provides a relatively hydrophobic environment into which an apolar pollutant can be trapped [5,6], through various kinds of interactions (van der Waals force, hydrophobic interaction, electrostatic affinity, dipole–dipole interaction and hydrogen bonding) [7–9]. CDs have been applied in various applications, such as chemical separations [10,11], as adsorbents [12,13], food processing [14] and as pharmaceutical excipients [15].

The contamination of water sources with phenols is increasing nowadays as a result of industrial and agricultural activities. Owing to their toxicity and carcinogenic properties, the presence of phenols in water can cause a reduction in water quality, decrease the number of aquatic organisms and also inhibit the common actions of biological communities. Therefore, phenol and its compounds have been listed as priority pollutants by the United States Environmental Protection Agency (US-EPA). Owing to their stability and bioaccumulation effects, effluents containing phenol and its derivatives must be treated prior to their release into water resources. There are various chemical and physiochemical methods including chemical oxidation, ion exchange, adsorption and membrane technology that have been proposed to eliminate phenolic compounds from polluted waters [16]. Nevertheless, the adsorption process is presently being used widely [17,18] because this procedure is the easiest, fastest, most efficient and cost-effective option for the removal of phenolic compounds [19].

In this study, βCD has been cross-linked with aromatic linker 2,4-toluene diisocyanate (2,4-TDI) and aliphatic linker 1,6-hexamethylene diisocyanate (1,6-HDI) to form insoluble βCD-TDI and βCD-HDI as sorbents for the removal of 2,4-dinitrophenol (DNP) from environmental water samples. Geometrically, studies reported that the hydrophobic nitro groups of 2,4-DNP are easily encapsulated into the βCD cavity and the resulting inclusion complexes can be stabilized by hydrogen bonding between the hydroxyl groups of 2,4-DNP and those of βCD [20,21].

The present work will investigate experimentally the sorption behaviours and capacities of both polymers. The influence of several batch parameters such as initial concentration, contact time, ionic strength, pH, sorbent dosage and initial temperature will be examined. The adsorption isotherm and kinetics of 2,4-DNP onto both polymers will be studied also, to identify the possible mechanism of the sorption process. Secondly, the investigation also allows us to ascertain the role and effect of cross-linking networks on adsorption of 2,4-DNP onto the βCD polymers.

## 2. Experimental procedure

### 2.1. Reagents and materials

βCD is commercially available and was purchased from Acros (Acros, Geel, Belgium) (99%). 2,4-TDI (95%), 1,6-HDI, dimethylformamide (DMF), buffer solution (pH 7.0) and acetonitrile were purchased from Sigma Aldrich and other chemicals used were analytical grade and were used without having to undergo further purification. Ultrapure water was used during the experiment. All the reactions were performed under inert conditions. 2,4-Dinitrophenol (2,4-DNP) was purchased from Sigma Aldrich (Steinheim, Germany). The structure and the properties of the studied phenol are shown in table 1. The standard stock solution of 2,4-DNP (1000 mg l^−1^) was prepared separately in acetonitrile and was stored in dark amber glass at 4°C to prevent degradation. The working solution was freshly prepared by diluting the stock solutions with deionized water.

**Table 1.** Structure and properties of the studied phenol.

analytes	chemical structure	dipole moment (debye)	log *K*_ow_	pK_a_
2,4-DNP	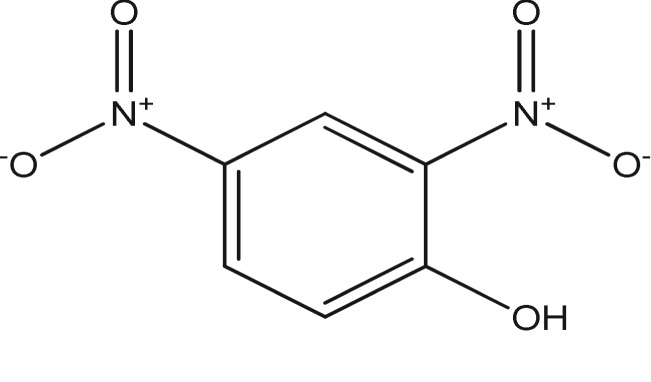	3.51	1.53	4.09

### 2.2. Synthesis and characterization

#### 2.2.1. Synthesis of insoluble β-cyclodextrin-toluene diisocyanate and β-cyclodextrin-hexamethylene diisocyanate polymer

**Figure 1.**
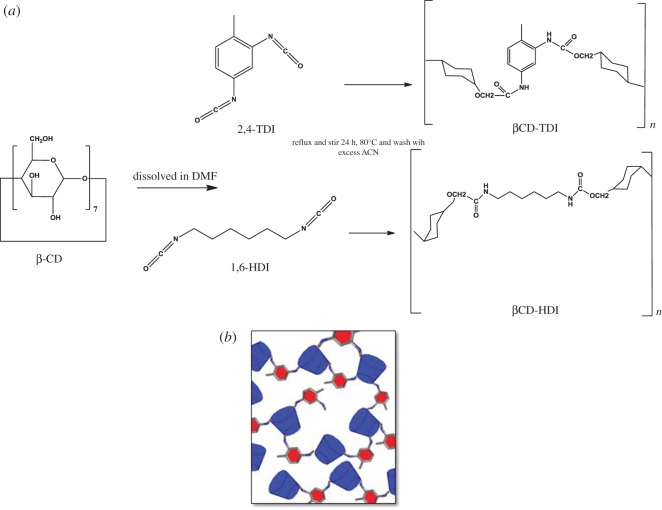
(*a*) Synthesis routes of βCD polymer from βCD; (*b*) Schematic of the βCD polymer structure.

βCD polymer was synthesized according to the reported method [22]. Two grams of βCD were dissolved with 40 ml of DMF at room temperature. Ten equivalents of cross-linker 1,6-HDI or 2,4-TDI was added dropwise into the mixture. Stirring was continued for a further 24 h at 80°C. The polymerization was monitored by infrared spectroscopy. The completion of the polymerization was confirmed by the total disappearance of the isocyanate peak at 2270 cm^−1^. After 24 h, the reaction mixture was then precipitated by the addition of acetone and the solid formed was allowed to settle in acetone for 10 min to remove the residual DMF from the polymer, followed by filtration, then was washed with acetone and double distilled water to remove the non-reactive cross-linker, and subsequently dried overnight under reduced pressure. The synthesis method is shown in figure 1.

#### 2.2.2. Characterization of the polymer

The Fourier transform infrared (FTIR) spectra were recorded on a Perkin Elmer Spectrum model spectrometer (Perkin Elmer, Waltham, MA, USA) between 4000 and 400 (cm^−1^) with a resolution of 2 cm^−1^. Meanwhile, the thermogravimetric analysis (TGA) curves were examined using a Q500 TGA instrument (Perkin Elmer). A linear heating rate was set at 20°C min^−1^ within the temperature range from 50 to 900°C in a stream of nitrogen atmosphere. Brunauer–Emmett–Teller (BET) analysis was determined from low-temperature nitrogen adsorption isotherms at 77.40 K using the Quantachrome Autosorb Automated Gas Sorption System (Quantachrome, Boynton Beach, FL, USA). Typically, at least 1 g of sample was used each time during the analysis. The surface area was obtained by the BET method, while the average pore diameter and pore volume of βCD-TDI and βCD-HDI in the solid dry state was measured from the adsorption branch of the isotherms by the Barret–Joyner–Halenda model. Apart from that, scanning electron microscope (SEM) analysis was used to obtain the morphology of the samples in micrographs with a Lecia S440 model (Leica, Germany) and magnification of 10 Kx for βCD-TDI and βCD-HDI.

### 2.3. Batch adsorption experiments

**Figure 2.**
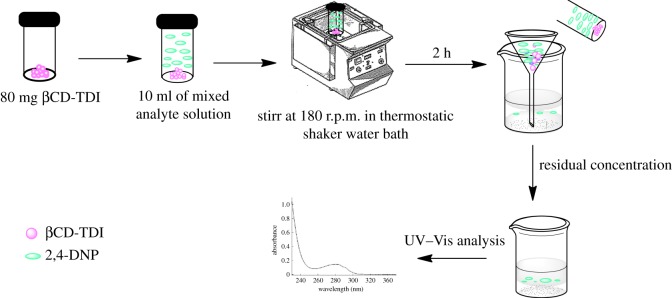
Schematic illustration of the βCD polymer for the removal of 2,4-DNP.

Batch experiments were carried out to investigate the parametric effects of contact time, pH, temperature and initial concentration of 2,4-DNP for adsorption onto both synthesized polymers. The experiments were carried out using 80 mg of each polymer in 10 ml of analyte aqueous solution fortified at a concentration of 10 mg l^−1^ in a tightly sealed vial. The solution was agitated at 180 r.p.m. in a thermostatic shaker water bath for 2 h. The adsorbent was separated by filtration after the adsorption process, and the residual concentration was determined separately using a UV–Vis Lambda-35 model spectrophotometer (Perkin Elmer) equipped with 1 cm quartz cells for 2,4-DNP at 358 nm wavelength. The pH of the solution was adjusted using an ST 3100-B pH meter (OHAUS, Shanghai, China). Removal procedures are shown in figure 2.

The removal efficiency of the studied analyte was calculated by the following equation:

2.1 $$R\;(\text{\%})=\frac{C_{e}-C_{o}\;}{{\text{C}}_{o}}\times \;100.$$

and in addition, the adsorption capacity (*q*_e_) was calculated according to the following equation:

2.2 $$q_{e}=\frac{(C_{o}-C_{e})V\;}{W},$$

where *C*_o_ and *C*_e_ are the initial and equilibrium concentrations of solutions (mg l^−1^), respectively. *V* (l) is the volume of the solution, and *W* (g) is the mass of the dry adsorbent used.

### 2.4. Adsorption kinetics

#### 2.4.1. Adsorption kinetic models

Several kinetic models were used to investigate the mechanism of adsorption and kinetic parameters, such as the pseudo-first order, pseudo-second order, Elovich, external diffusion and intraparticle diffusion models. The characteristics and equations of these models [14] are described in detail below.

*Pseudo-first-order model.* The pseudo-first-order kinetic model is given by the following equation:

2.3 $$\log(q_{e}-q_{t})=\log q_{e}-\;\frac{K_{1}t}{2.303},$$

where *t* is the contact time, *q*_e_ and *q*_t_ are the amount of analyte adsorbed (mg g^−1^) at equilibrium and at contact time, respectively, and *K*_1_ is the rate constant of this equation (min^−1^). The values of *K*_1_ and *q*_e_ were calculated from the plot of log (*q*_e_ − *q*_t_) versus *t*.

*Pseudo-second-order model.* The sorption data were also analysed in terms of the pseudo-second-order model as:

2.4 $$\frac{t}{qt}=\frac{1}{K_{2}qe^{2}}+\;\frac{1t}{q_{e}},\;h=\;K_{2}qe^{2}\;\;\text{and}\;\;t_{1/2}=(Kqe^{-1}),$$

where *h* is defined as the initial adsorption rate (mg g^−1^) min, *t*_1/2_ the half-equilibrium time (min) and *K*_2_ the pseudo-second-order rate constant (g mg^−1^ min). The values of *q*_e_, *K*_2_, *h* and *t*_1/2_ were obtained by a linear plot of *t*/*q*_t_ versus *t*.

*Elovich model.* The Elovich model is generally defined as:

2.5 $$q_{t}=\;\frac{1}{\beta }\ln(\alpha \beta )+\;\frac{1}{\beta }\ln t,$$

where *α* is the initial sorption rate (mg g^−1^ min^−1^), and *β* is related to the extended surface coverage and activation energy for chemisorption (g mg^−1^). The values of *α* and *β* can be obtained by a linear plot of *q_t_* versus ln *t*.

#### 2.4.2. Validation of kinetic models

The suitability of the model to describe the adsorption kinetics was further justified and predicated on the normalized standard deviation value, Δ*q* (%) and relative error (%) which is defined as in equations (2.6) and (2.7):

2.6 $$\Delta q\;(\text{\%})=\sqrt{\frac{{\left[(q_{\exp}-q_{\mathrm{cal}})/q_{\exp}\right]}^{2}\;}{N-1}\;}\times 100$$

and

2.7 $$\text{relative error}(\text{\%})=\frac{\left|qe_{\mathrm{cal}}-qe_{\exp}\right|}{qe_{\exp}}\times 100.$$

#### 2.4.3. Adsorption mechanism

The adsorption mechanism was studied to further investigate and understand the adsorption process and to determine the rate of the controlling step, which mainly depends on the external diffusion/film, followed by the intraparticle or pore diffusion, and finally, the sorption into the interior sites of the adsorbent. The sorption of the adsorbate on the sorbent may be governed by external and/or intraparticle diffusion because the final process is very rapid.

*External diffusion.* The external diffusion model or film diffusion is described as follows:

2.8 $$\ln\frac{C_{t}}{C_{o}}=\;-K_{\mathrm{ext}}t,$$

where *C*_o_ and *C_t_* represent the concentration of the solute in the initial solution and in the liquid phase at time *t*, respectively, and *K*_ext_ (min^−1^) is a diffusion rate parameter. The plot of ln (*C_t_*/*C*_o_) against *t* should give a linear line with zero intercept if the external diffusion is applicable.

*Weber and Morris model.* The intraparticle diffusion model can be expressed as:

2.9 $$q_{t}=Kt^{0.5}+c,$$

where *c* represents the intercept (mg g^−1^), and *K* the intraparticle diffusion rate constant (mg g^−1^ min^−1^).

If the adsorption of phenol compounds on βCD-TDI fits the intraparticle model, a plot of *q_t_* versus *t*^0.5^ (square root of time) should be linear, and when this line passes through the origin the intraparticle diffusion is said to be the rate-controlling step. Some degrees of boundary layer control further prove that the intraparticle diffusion is not the only rate-limiting step, while other kinetic models may control the rate of adsorption, all of which may be operated simultaneously if the plot does not pass through the origin.

### 2.5. Adsorption equilibrium

#### 2.5.1. Langmuir model

The Langmuir equation was used to estimate the maximum adsorption capacity that is related to complete monolayer coverage on the adsorbent surface and is expressed by the following equation [14]:

2.10 $$\frac{1}{q_{e}}=\;\frac{1}{bq_{m}}+\;\frac{C_{e}}{q_{m}},$$

where *C*_e_ (mg l^−1^) is defined as the equilibrium concentration of the adsorbate, *q*_e_ (mg g^−1^) is the adsorption capacity at equilibrium, and *q*_m_ (mg g^−1^) and *b* (l mg^−1^) are Langmuir constants that are related to the adsorption capacity and the rate of adsorption, respectively.

#### 2.5.2. Freundlich model

The Freundlich isotherm was used to estimate the adsorption intensity of the sorbent towards the adsorbate and it is assumed that it serves as a heterogeneous system with different energies of active sites and reversible adsorption. The linear form of the Freundlich isotherm can be expressed as follows [14]:

2.11 $$\log q_{e}=\log K_{F}+\;\frac{1}{n}\log C_{e}.$$

The Freundlich constants can be obtained from the plot of log *q*_e_ versus log *C*_e_: *K*_F_ ((mg g^−1^)(l mg^−1^)^1/*n*^) indicates the adsorption capacity, whereas *n* values indicate the favourability of the adsorption process. If *n* is above unity, then the adsorption process is favourable.

### 2.6. Adsorption thermodynamics

The data obtained from the temperature studies were used for thermodynamic analysis. The standard free energy change of the sorption reaction is given by equation (2.12), while the enthalpy change (Δ*H*°) and entropy change (Δ*S*°) were calculated from the slope and intercept of the Van't Hoff plot, respectively (ln *K*_d_ versus 1/*T*) using equation (2.13) [23]:

2.12 $$\Delta G^{o}=\;-RT\ln\;K_{d}$$

and

2.13 $$\ln\;K_{d}=\;\frac{\Delta S^{o}}{R}-\;\frac{\Delta H^{o}}{RT},$$

where *R* represents the ideal gas constant (8.314 J mol^−1^ K^−1^), *T* is the temperature (*K*) and *K*_d_ the standard thermodynamic equilibrium constant.

### 2.7. Preparation of environmental water samples

Water samples were collected in disposable 500 ml polypropylene bottles. Bottles were pre-cleaned with ultrapure water and methanol and baked at 110°C for 3 h prior to use. Tap water (from Integrative Medicine Cluster laboratory, Universiti Sains Malaysia), lake water (from Bukit Panchor State Park, Penang, Malaysia), river water (from Sungai Buaya, Nibong Tebal, Penang, Malaysia) and sea water (from Penang island, Malaysia) were collected between March and May 2017. Bottles were filled completely up to the rim to eliminate the headspace. Samples were filtered and stored at 4°C prior to processing. Analyses were carried out within two weeks of the sample collection. Samples were used in their original condition without any pH and temperature adjustment, or dilution.

## 3. Results and discussion

### 3.1. Characterization of β-cyclodextrin-toluene diisocyanate and β-cyclodextrin-hexamethylene diisocyanate

#### 3.1.1. Fourier transform infrared analysis

**Figure 3.**
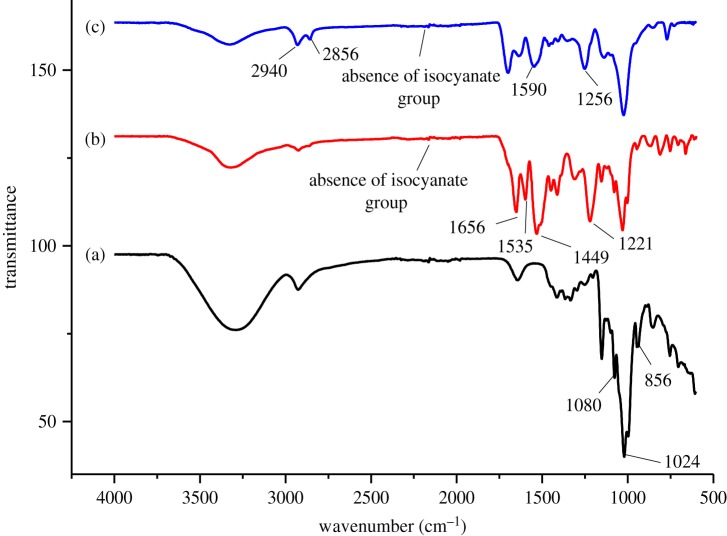
FTIR spectra of (a) βCD, (b) βCD-TDI and (c) βCD-HDI.

The chemical structures of βCD-TDI and βCD-HDI were analysed by FTIR spectrometry and their FTIR spectra were compared with native β-CD as shown in figure 3. In the spectrum of β-CD, the peak at 856 cm^−1^ corresponds to α-(1,4) glucopyranose, whereas the peaks at 1024 and 1080 cm^−1^ were contributed by the C–OH stretching vibration. The absence of a peak at 2270 cm^−1^ (corresponding to the isocyanate group) in figure 3*b* and *c* indicated that the polymerization reaction was complete. βCD-TDI showed peaks at 1449 and 1656 cm^−1^, suggesting the presence of the aromatic group of the TDI cross-linker. Meanwhile, the peaks at 2856 and 2940 cm^−1^ correspond to the methylene group of the HDI cross-linker. Furthermore, the stretching vibration of NHCO, which indicates the formation of a carbamate group during the polymerization process, was observed at 1535 cm^−1^ in figure 3*b* and at 1590 cm^−1^ in figure 3*c*. The adsorption bands at 1221 and 1256 cm^−1^ could be attributed to the C–O–C characteristics of polyurethane. These results confirmed the successful polymerization between βCD molecules and both cross-linkers. The detailed band assignments related to the FTIR analyses of the βCD, βCD-TDI and βCD-HDI are given in table 2.

**Table 2.** Details of band assignments related to the FTIR analyses of βCD, βCD-TDI and βCD-HDI.

samples	wavelength (cm^−1^)	assignments
βCD	856.1	α-(1,4)glucopyranose
	1024, 1080	C–OH stretching vibration
βCD-TDI	1221	C–O–C of polyurethane
	1449	aromatic group in TDI
	1535, 1656	NHCO, carbamate linkage
	2270	absence of isocyanate group
βCD-HDI	1256	C–O–C of polyurethane
	1590	NHCO, carbamate linkage
	2270	absence of isocyanate group
	2940, 2856	CH_2_ asymmetric stretching vibration in HDI

#### 3.1.2. Thermogravimetric analysis

**Figure 4.**
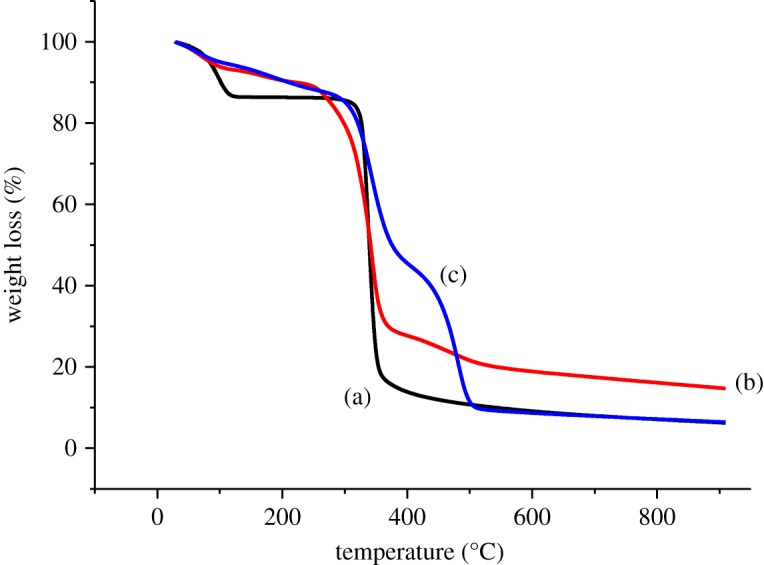
TGA curve of (a) βCD, (b) βCD-TDI and (c) βCD-HDI.

TGA was performed to quantitatively characterize the polymerization process and the thermal stability between βCD and the cross-linked polymers. From figure 4, native βCD started to decompose at 120°C. Visible changes occurred at 320°C which corresponded to the loss of water molecules in the βCD cavity.

Meanwhile, the weight loss of both βCD-TDI and βCD-HDI was mainly divided into two temperature regions: below 140°C and around 120–480°C. The weight loss during the heating below 140°C was assigned to the loss of physically adsorbed water. The higher weight loss around 120–480°C was likely due to the decomposition of the attached carbamate groups contributed by the TDI and HDI cross-linkers on βCD, suggesting the successful preparation of the polymers.

In copolymer composition, the cross-linker is attributed to the thermal properties of a polymer. In most cases, rigid and aromatic ring containing polymers are more stable towards thermal action, whereas aliphatic and flexible cross-linker containing polymers are less stable towards thermal action [24]. From the TGA analysis, βCD-TDI exhibited higher stability compared to βCD-HDI because it showed a lower weight loss. This could be explained by the different structures of the cross-linkers used because TDI is an aromatic cross-linker which possesses a strong electrostatic interaction between the conjugated *π* systems, while HDI is only an aliphatic cross-linker without forming any *π* to *π* interaction. The degradation and weight loss steps of βCD and the βCD polymers are shown in table 3.

**Table 3.** Degradation and weight loss steps of βCD, βCD-TDI and βCD-HDI.

sample	region (°C)	weight loss (%)	assignment
βCD	120	14.0	water loss
	320–410	87.0	βCD
βCD-TDI	140	7.3	water loss
	120–480	77.0	βCD and C=O
βCD-HDI	140	7.0	water loss
	120–480	80.2	βCD and C=O

#### 3.1.3. Scanning electron microscope analysis

Microscopic morphological structures were examined using SEM in order to determine and compare the surface features of native βCD, βCD-TDI and βCD-HDI. Basically, the pore formation depends on the chemical structure of the polymer backbone, the polymerization method, the stirring speed and the type of cross-linker (hydrophobic or hydrophilic) [24,25].

**Figure 5.**
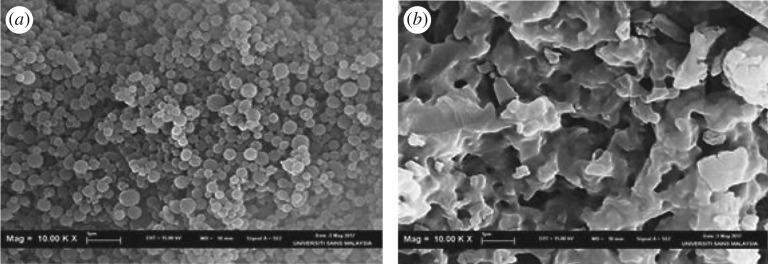
SEM image of (*a*) βCD-TDI and (*b*) βCD-HDI.

SEM images of βCD-TDI and βCD-HDI are presented in figure 5. βCD-TDI exhibited monodispersed small crystalline structure with well-arranged spherical and smooth surfaces. This is due to the fact that the hydrophilic TDI cross-linker has a greater affinity towards the aqueous phase which allows a decrease in the polymer particle size in a homogenous form.

In comparison to this morphology, βCD-HDI showed a ‘shrinking’-like crystalline structure with a fluffy and relatively coarse surface resulting from the hydrophobic HDI cross-linker. The HDI cross-linker favoured the organic phase, producing a non-uniform surface for the polymer as shown in figure 5*b*.

#### 3.1.4. Brunauer, Emmett and Teller analysis

**Figure 6.**
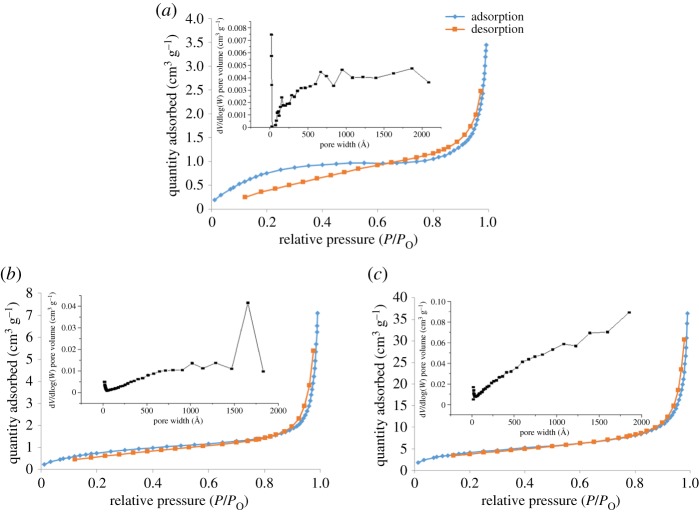
Nitrogen adsorption–desorption isotherms of (*a*) βCD, (*b*) βCD-TDI and (*c*) βCD-HDI.

The surface properties of both βCD-TDI and βCD-HDI polymers were investigated by nitrogen physisorption measurements and compared with native βCD. The corresponding adsorption–desorption isotherms are displayed in figure 6. Their adsorption–desorption isotherms exhibited a type IV isotherm with H3 type hysteresis loop at a relative pressure between 0.0 and 1.0, characteristic for mesopores with a regular pore size distribution of 15.5 and 17.2 nm for βCD-TDI and βCD-HDI, respectively. The surface area of both polymers were calculated using the BET equation. It was found that βCD-TDI (2.5 m^2^ g^−1^) exhibited a lower surface area than βCD-HDI (14.0 m^2^ g^−1^). Meanwhile, native βCD represented a micropore structure with pore size of 1.58 nm and surface area of 2.45 m^2^ g^−1^.

βCD-TDI exhibited lower surface area compared to βCD-HDI in the solid dry state. This phenomenon is most probably due to the effect of the cross-linkers used. In general, aliphatic diisocyanates are less susceptible to water than the aromatic diisocyanates [26], thus aliphatic diisocyanate (HDI) is a hydrophobic cross-linker that possesses higher surface area and aromatic diisocyanate (TDI), which is a hydrophilic cross-linker, possesses lower surface area.

Therefore, with the analysis based on BET, it is reasonable to believe that the high surface area of βCD-HDI is due to the hydrophobic cross-linker that shows more affinity towards the organic phase, which give rise to a mesoporous architecture. This finding was in good agreement with a few other studies [24,27].

### 3.2. Optimization of adsorption parameters

#### 3.2.1. Effect of initial pH

**Figure 7.**
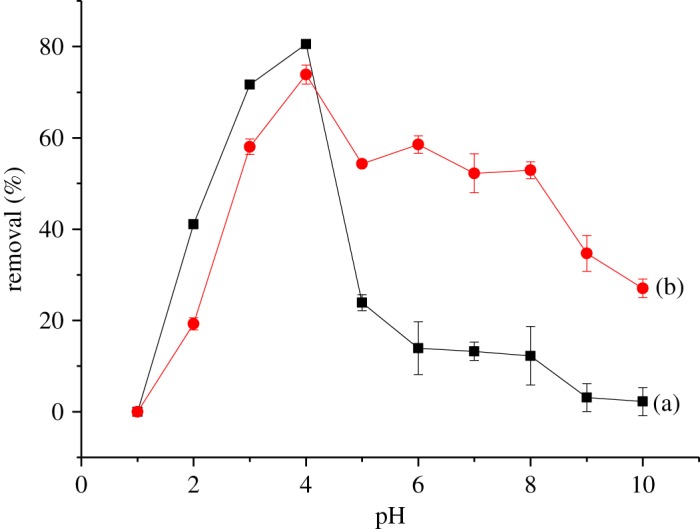
Effects of initial pH on the removal efficiency of 2,4-DNP by (a) βCD-TDI and (b) βCD-HDI (condition: sorbent, 20 mg; initial concentration of 2,4-DNP, 10 mg l^−1^; volume, 10 ml; time, 120 min; temperature, 298 K).

Solution pH plays an important role in the adsorption studies as it controls both degree of ionization of the materials present in the solution and the dissociation of functional groups of the analytes. The initial pH of the solution was adjusted to the desired value from 2 to 10 either by the addition of HCl or NaOH. Results revealed that the adsorption was strongly pH-dependent. As shown in figure 7, the best result was acquired when the pH value was 4 for both βCD-TDI and βCD-HDI and gradually decreased as the pH increased from 5 to 10. This phenomenon could be attributed to the deprotonation of the phenolic hydroxyl groups in a high pH medium that converted them into phenoxide ions (RO^−^, R = aromatic ring). Following the deprotonation as well as the high abundance of OH^−^ ions, the removal efficiency was reduced as a result of the competition between high concentrations of hydroxyl groups with the phenol molecules [28]. Besides, deprotonated forms of 2,4-DNP were not in favour of forming a stable complex in the cavity of βCD in a high pH medium [29].

In low pH solution, more protons (H^+^) would be available to protonate 2,4-DNP (pKa = 3.96) molecules; however, the protonation of 2,4-DNP was very difficult to achieve because it needed very strong acidic condition (pH 1 or pH 2) and phenol compounds preferred to be in the molecular form when pH < pKa, thus leading to a low removal ability of both polymers towards 2,4-DNP.

**Figure 8.**
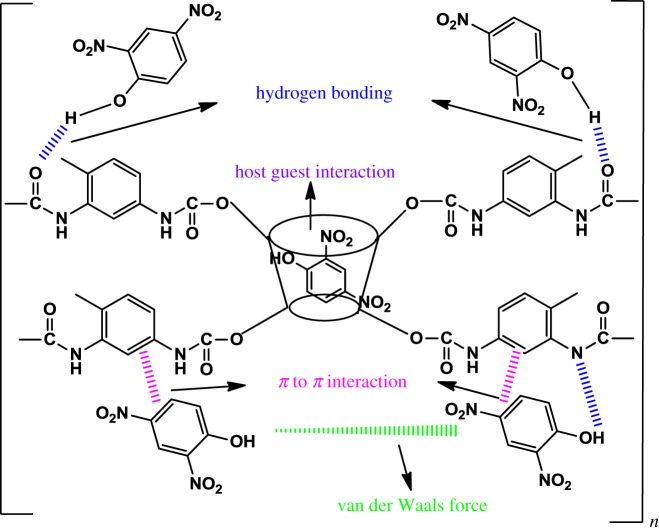
Proposed possible adsorption mechanisms for 2,4-DNP using βCD-TDI.

The sorption mechanism of both polymers with 2,4-DNP is mainly through van der Waals forces, hydrogen bonding and π to π stacking interaction (as shown in figure 8). In addition to this, because βCD molecules have a remarkable capacity to form inclusion complexes with phenols [30,31], in the present study, host guest interaction could also occur as well as other interactions because the cavity of βCD is maintained during the polymerization process.

By considering the highest removal efficiency of 2,4-DNP, pH 4 was selected for further experiments. However, the removal efficiency of βCD-TDI (80.56%) was higher than that of βCD-HDI (73.87%). This could be explained by the effects of the cross-linker used, because TDI is an aromatic cross-linker that can form stronger π to π stacking interaction due to its conjugated aromatic system with 2,4-DNP rather than HDI which possesses only the aliphatic backbone [32]. The aromatic TDI cross-linker showed higher reactivity towards 2,4-DNP because it has a similar structure to 2,4-DNP. Thus, it behaves in a similar way, leading to the higher results obtained.

#### 3.2.2. Effect of contact time

**Figure 9.**
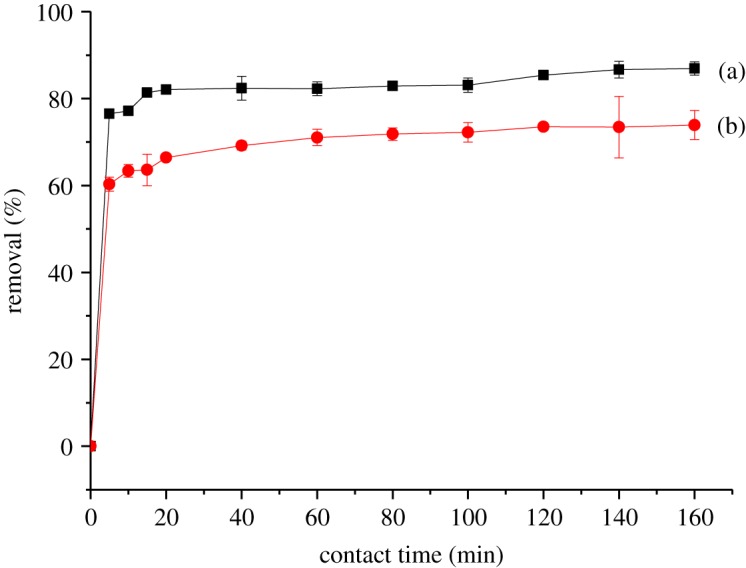
Effects of initial time on the removal efficiency of 2,4-DNP by (a) βCD-TDI and (b) βCD-HDI (condition: sorbent, 20 mg; initial concentration of 2,4-DNP, 10 mg l^−1^; volume, 10 ml; temperature, 298 K; pH, 4.0).

According to the literature, contact time is a fundamental parameter in the adsorption study. The effect of contact time on the adsorption of 2,4-DNP was investigated using βCD-TDI and βCD-HDI in the range of 0–160 min. The removal percentage of 2,4-DNP by both polymers as a function of contact time is presented in figure 9. As is shown, maximum percentages of removal were achieved at 120 min and thereafter become constant, achieving equilibrium. From the results, the removal efficiency increased sharply as the contact time increased from 0 to 20 min. The major reason behind this is that initially all of the active sites on the adsorbent surface were unoccupied, but after time, the site becomes occupied with the analyte and only a few surface active sites remain available for further adsorption, resulting in the saturation of the adsorption capacity. Thus, the percentages of removal remain constant.

Although the optimum contact time for both polymers was achieved at 120 min, the removal ability of βCD-TDI (85.4%) towards 2,4-DNP was higher compared to βCD-HDI (73.5%). Therefore, to obtain better removal efficiency, 120 min was selected for further study for both of the polymers.

#### 3.2.3. Effect of 2,4-dinitrophenol initial concentration

**Figure 10.**
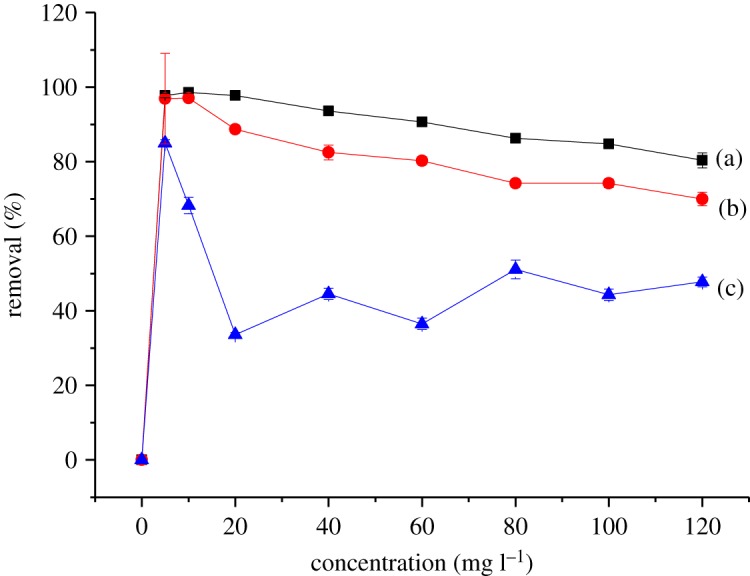
Effects of initial concentration on the removal efficiency of 2,4-DNP by βCD-TDI at (a) 298 K, (b) 318 K and (c) 338 K (condition: sorbent, 20 mg; volume, 10 ml; time, 120 min; pH, 4.0).

**Figure 11.**
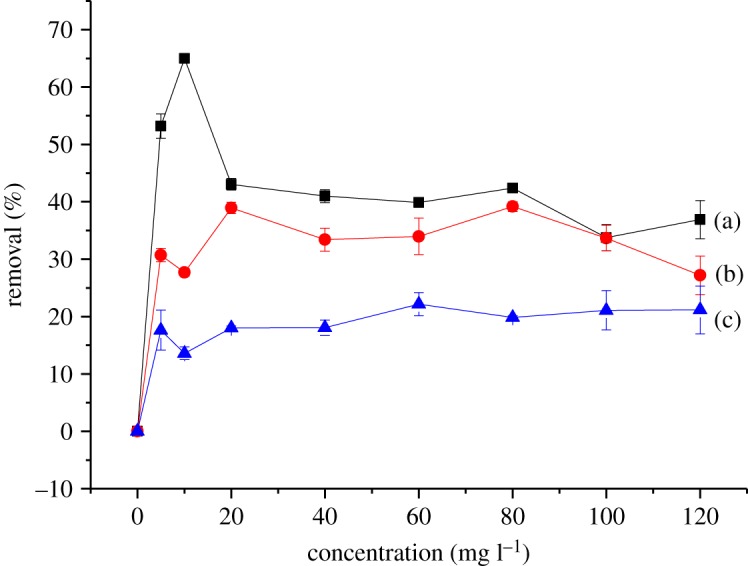
Effects of initial concentration on the removal efficiency of 2,4-DNP by βCD-HDI at (a) 298 K, (b) 318 K and (c) 338 K (condition: sorbent, 20 mg; volume, 10 ml; time, 120 min; pH, 4.0).

Figures 10 and 11 show the effect of the initial concentration of 2,4-DNP in the range of 5–120 mg l^−1^ on βCD-TDI and βCD-HDI at 298, 318 and 338 K, respectively. The initial concentration provided an important driving force to overcome all the mass transfer resistance of 2,4-DNP between the aqueous and solid phases of the adsorbents [33]. Besides, the initial concentrations of 2,4-DNP in solution dominate the theoretical maximum adsorbed amounts, and affect the actual adsorbed amounts on both polymers.

High adsorption efficiency observed at a concentration of 10 mg l^−1^ could be due to the availability of more active sites on the adsorbent than the numbers of phenol ions in the solution, while at higher concentration, phenol ion numbers are more than the active sites of the adsorbent leading to a limited capacity of the polymer [34].

Apart from that, the complexation between 2,4-DNP and the βCD molecule was also influenced by the molecular structure and hydrophobicity of the 2,4-DNP compound [35]. In this study, 2,4-DNP had loss of hydrophobicity at high concentration and it was unable to form a stable complex with the cavity of βCD, causing a reduction in the adsorption capacity for both polymers.

#### 3.2.4. Effect of solution temperature

**Figure 12.**
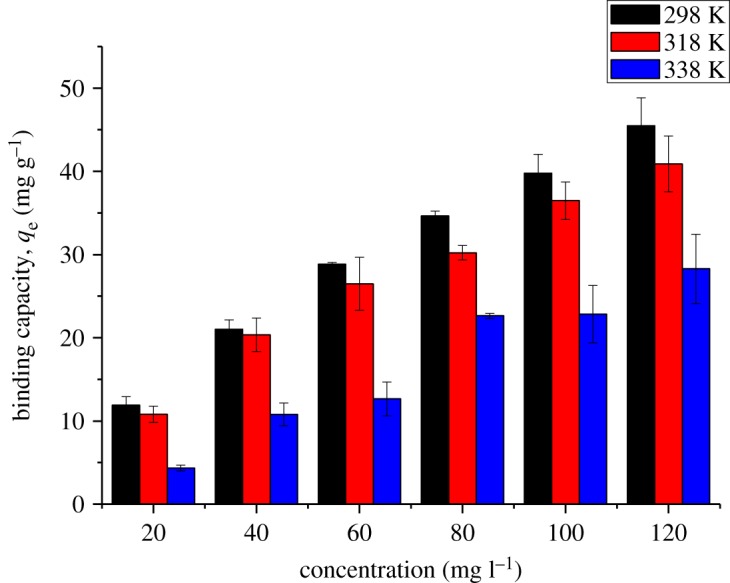
Effects of solution temperature on the binding capacity of 2,4-DNP by βCD-TDI (condition: sorbent, 20 mg; volume, 10 ml; time, 120 min; initial concentration of 2,4-DNP, 10 mg l^−1^; pH, 4.0).

**Figure 13.**
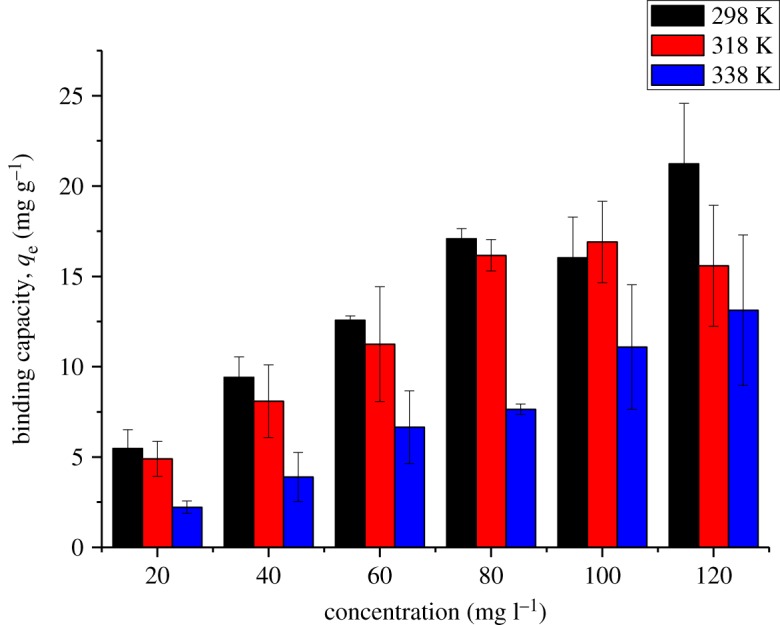
Effects of solution temperature on the binding capacity of 2,4-DNP by βCD-HDI (condition: sorbent, 20 mg; volume, 10 ml; time, 120 min; initial concentration of 2,4-DNP, 10 mg l^−1^; pH, 4.0).

The solution temperature was optimized because heat plays a role in affecting the binding capacity of analyte on the active sites of adsorbents. As expected, herein, the binding capacity, *q*_e_ (mg g^−1^), was found to decrease as the temperature increased. This could be due to the exothermic reaction which distorted the binding sites of the analytes leading to poor binding interaction between the 2,4-DNP molecule and adsorbent, and as a consequence, the removal efficiency decreased. By considering the optimum binding ability of 2,4-DNP on both polymers, the experiments were carried out at 298 K. Figures 12 and 13 illustrate the binding capacity of 2,4-DNP onto βCD-TDI and βCD-HDI polymers at different temperatures, respectively.

#### 3.2.5. Effect of sorbent dosage

**Figure 14.**
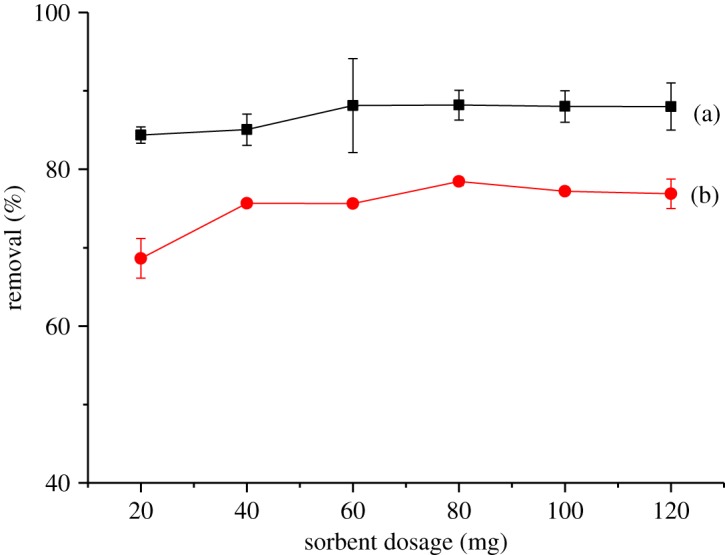
Effects of sorbent dosage on the removal efficiency of 2,4-DNP by (a) βCD-TDI and (b) βCD-HDI (condition: initial concentration of 2,4-DNP, 10 mg l^−1^; volume, 10 ml; time, 120 min; temperature, 298 K; pH, 4.0).

The optimization of adsorbent dose is also an important parameter in adsorption studies because it determines the adsorption capacity of an adsorbent for a given initial concentration of analyte in solution. For this, different amounts of βCD-TDI and βCD-HDI were applied. Figure 14 shows the effect of adsorbent dosage on the removal efficiency. Initially, the 2,4-DNP removal efficiency increased sharply as the adsorbent dosage increased from 20 to 60 mg owing to the increase in the number of available adsorption sites, and the highest removal was achieved at 80 mg of the sorbents. In addition, the highest phenol removal efficiency was obtained by βCD-TDI which could be attributed to the interaction of 2,4-DNP with the aromatic ring structure present in the TDI cross-linker, when compared with the HDI cross-linker.

There was no obvious variation in the removal percentages of 2,4-DNP with a further increase in the sorbents after that, thus some active sites remain unsaturated and the adsorption process reaches an equilibrium. Therefore, 80 mg was selected for the entire study as the equilibrium point, and it was used for the subsequent analysis.

#### 3.2.6. Effect of ionic strength

**Figure 15.**
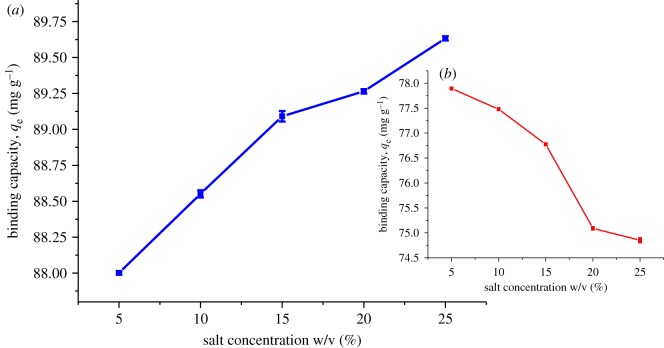
Effects of salt addition on the binding capacity of 2,4-DNP by (*a*) βCD-TDI and (*b*) βCD-HDI (condition: sorbent, 80 mg initial concentration of 2,4-DNP, 10 mg l^−1^; volume, 10 ml; time, 120 min; temperature, 298 K; pH, 4.0).

In order to further enhance the partitioning of targeted analytes onto the polymers, the ionic strength of the sample solution was examined by adding NaCl at concentrations of 5, 10, 15, 20 and 25% (w/v) to the sample solution. The effect of salt addition is shown in figure 15. As is shown, the binding capacity of 2,4-DNP onto βCD-TDI increased gradually as the concentration of salt increased, most probably due to the salting-out effect. Salting-out effect is a phenomenon in which the increase of salt will reduce the solubility of analyte in the aqueous phase [36]. Thus, this phenomenon facilitates the mobility of the targeted analytes towards the polymer. Salt ions in a sample solution may penetrate into the diffuse double layer and significantly eliminate the repulsive energy between adsorbents [37,38], and implicitly enhance the polymer adsorption against the targeted analytes. Thus, it is reasonable that βCD-TDI showed better results in the presence of salt, besides the π–π interaction that exists between the TDI cross-linker and 2,4-DNP aromatic ring.

However, experimental data revealed that salt addition had a slightly negative effect on the binding capacity of 2,4-DNP by βCD-HDI. In this case, the sample solution becomes more viscous with the addition of salt [39], which resulted in difficult mass transfer and lower binding capability of 2,4-DNP by βCD-HDI. The addition of salt had reduced the interaction of 2,4-DNP with the βCD-HDI polymer surface and created a competition between Na^+^ cations and DNP, but not with the βCD polymer.

Therefore, 25% (w/v) of added salt was selected for βCD-TDI polymer and no salt was added to the solution in the subsequent analysis for βCD-HDI.

### 3.3. Sorption kinetics

To further investigate the adsorption mechanism and its potential rate-controlling steps such as chemical reaction, mass transfer and diffusion control, the adsorption parameters derived from the pseudo-first-order (*K*_1_ and *q*_e_) and second-order kinetics models (*K*_2_, *q*_e_), intraparticle diffusion (*K_i_*), external diffusion and Elovich model (*α* and *β*) were calculated and listed in table 4.

**Table 4.** Kinetic parameters for 2,4-DNP adsorption onto βCD-TDI and βCD-HDI.

kinetic models	parameters	βCD-TDI	βCD-HDI
pseudo-first-order kinetic model	*q*_e_,exp (mg g^−1^)	3.954	3.469
	*q*_e_,cal (mg g^−1^)	0.041	0.166
	*K*_1_ (min^−1^)	0.022	0.042
	Δ*q* (%)	31.290	30.110
	relative error (%)	98.960	95.220
	*R*^2^	0.604	0.916
pseudo-second-order kinetic model	*q*_e_,cal (mg g^−1^)	3.895	3.438
	*K*_2_ (g mg^−1^ min^−1^)	0.027	0.090
	*t*_1/2_ (min)	8.850	3.120
	Δ*q* (%)	0.472	0.283
	relative error (%)	1.490	0.894
	*R*^2^	0.9993	0.9998
Elovich equation	*q*_e_,cal (mg g^−1^)	3.895	3.438
	*β*	0.169	5.227
	*α*	10.413	111829.14
	*R*^2^	0.8656	0.9875
intraparticle diffusion	*q*_e_,cal (mg g^−1^)	3.895	3.438
	*K_i_* (mg g^−1^ min^1/2^)	0.016	0.019
	*C* (mg g^−1^)	3.098	3.163
	*R*^2^	0.9666	0.9652
external diffusion	*C*	−407	−355
	*R*^2^	0.8434	0.8509

As shown in the table, for both polymers, it was observed that the *R*^2^ value of pseudo-second-order kinetic model (0.9993 and 0.9998 for βCD-TDI and βCD-HDI) is significantly higher than those of the pseudo-first-order kinetic model (0.6036 and 0.9155 for βCD-TDI and βCD-HDI) and Elovich model (0.8656 and 0.9875 for βCD-TDI and βCD-HDI). The pseudo-second-order model fit is better and is more precise, thus the pseudo-second model is the most recommended model for the description of the adsorption of phenol on both polymers and could be used to determine the equilibrium sorption capacity, rate constants and removal percentage of phenol. Likewise, the data suggested that most probably 2,4-DNP molecules were adsorbed onto the surface of βCD-TDI and βCD-HDI by the chemisorption mechanism, which involves the valence forces through the sharing or exchange of electrons, and the adsorption mechanism might depend on both the adsorbate and the adsorbent [40].

Basically, the adsorption kinetics is controlled by three steps related to the adsorption of solute from the solution by an adsorbent. These include (i) external diffusion/film diffusion, (ii) intraparticle/pore diffusion and (iii) sorption into interior sites [41]. The rate of the last step is very fast and considered to be negligible; hence, the overall rate of adsorption is controlled by the slowest step, which would either be external or internal diffusion [1,42].

**Figure 16.**
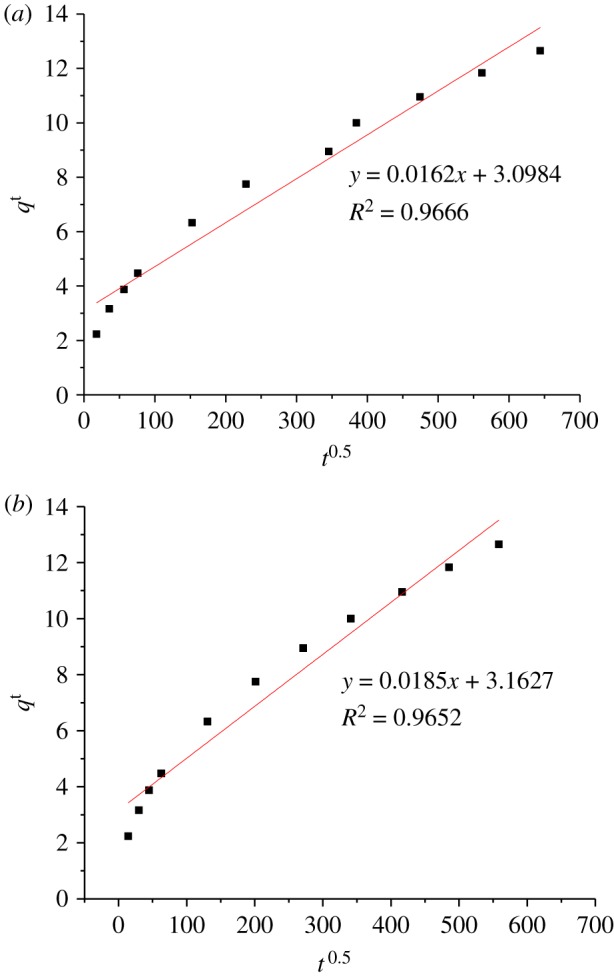
Linearized intraparticle diffusion kinetic model of 2,4-DNP sorption onto (*a*) βCD-TDI and (*b*) βCD-HDI.

The intraparticle kinetic model of 2,4-DNP sorption for the two polymers is shown in figure 16. The non-zero intercepts of the plots in each case indicated that the intraparticle diffusion is not the rate-controlling step for the sorption mechanism. In other words, the intraparticle diffusion model is not the only rate-determining step. The difference in the rate of mass transfer or the mass transfer resistance effect during the initial and final stages of adsorption could be the factor that led to the linearity deviation from the origin.

Figure 16 also demonstrates that the intraparticle diffusion of 2,4-DNP within the polymers happened in two phases as the plots contain two different line patterns. This could be explained by the rapid adsorption of 2,4-DNP to the external surface of polymers at the initial stage for both polymers, from 0 to 20 min. The second phase involves a gradually increasing adsorption from 20 to 25 min, corresponding to the slow intraparticle diffusion of the phenol molecules into the mesopore structure of the βCD polymers.

The external diffusion model was studied to investigate whether or not other mechanisms were involved. A linear plot with an *R*^2^ of 0.84 and 0.85 for βCD-TDI and βCD-HDI, respectively, and an intercept value of −407 and −355, suggested that the external diffusion is also not the only rate-limiting step in the adsorption process.

Therefore, these results suggested that intraparticle diffusion and external diffusion occurred simultaneously during the phenol adsorption process for both polymers.

### 3.4. Adsorption isotherm

In general, the adsorption isotherm analysis was performed to provide qualitative information about the adsorption capacity of adsorbent and distribution of adsorbate between solid and liquid phases, at the time of equilibrium. Therefore, the adsorption isotherm was studied at three different temperatures (298, 318 and 338 K) using the linearized forms of Langmuir and Freundlich isotherm models to describe the equilibrium nature of adsorption, and the isotherm constant is listed in table 5.

**Table 5.** Freundlich and Langmuir isotherm parameters for 2,4-DNP removal by βCD-TDI and βCD-HDI.

isotherms	βCD-TDI			βCD-HDI		
	temperature					
	298 K	318 K	338 K	298 K	318 K	338 K
Langmuir						
*q*_m_ (mg g^−1^)	−1.6978	−2.8596	−5.4025	222.22	294.12	−61.35
*b* (l mg^−1^)	−0.0340	−0.014	−0.072	0.0210	0.0360	−0.025
*R*^2^	0.9795	0.9171	0.7778	0.9882	0.9651	0.9010
Freundlich						
*K*_F_ ((mg g^−1^)(l mg^−1^)^1/*n*^)	10.129	4.5426	1.1819	0.0186	0.0101	0.6028
*n*	1.1455	1.0646	0.7356	4.9370	0.0001	0.6713
*R*^2^	0.9901	0.9714	0.9601	0.9872	0.9493	0.8373

From table 5, it can be seen from the correlation determination (*R*^2^) that the Langmuir model fits well for βCD-HDI, suggesting a monolayer coverage of 2,4-DNP molecules with a definite homogenous distribution on active sites of the polymer with uniform energies of adsorption, without the transmigration of the adsorbate in the plane of the polymer surface.

However, the Freundlich isotherm model works well for βCD-TDI, as can be seen from correlation determination, suggesting a multilayer coverage of 2,4-DNP molecules onto the polymers and also heterogeneous distribution of active sites and adsorption heat on the adsorbent.

Based on the data, it can be seen that the type of bifunctional isocyanate linker plays an important role to control the behaviour of adsorbate during the adsorption process. Table 5 also shows βCD-TDI has a higher adsorption capacity, while βCD-HDI has a lower adsorption capacity. This is most probably due to the fact that βCD-TDI consists of an aromatic ring and is able to entrap more 2,4-DNP molecules into the polymer network due to π to π interaction. Therefore, the capacity of βCD-TDI is higher than βCD-HDI.

In the Freundlich isotherm model, the value of ‘*n*’ indicates the degree of non-linearity between solution concentration and adsorption as follows: if *n* = 1, then adsorption is linear; if *n* < 1, then the adsorption is a chemical process; if *n* > 1, then the adsorption is a physical process [43,44]. The value of ‘*n*’ in the Freundlich equation was found to be 1.15 and 4.94 for βCD-TDI and βCD-HDI at 298 K (table 5), which suggests that physical adsorption of 2,4-DNP molecules occurs on the polymer surface.

### 3.5. Adsorption thermodynamics

The thermodynamic parameters obtained for the adsorption of 2,4-DNP are tabulated in table 6. Both polymers show negative values of −72.12 and −51.90 for enthalpy (Δ*H*°), indicating that the process was exothermic, which is in a good agreement with the previously calculated Freundlich constant (*K*_F_) that showed a decreasing trend as the temperature increased. Apparently, βCD-TDI has a higher value of Δ*H*° and reveals the presence of strong chemical bonds with 2,4-DNP.

**Table 6.** Thermodynamic adsorption parameters.

	thermodynamic parameters			
sorbent	Δ*H°* (kJ mol^−1^)	Δ*S*° (J mol^−1^ K^−1^)	*T* (K)	Δ*G*° (kJ mol^−1^)
βCD-TDI	−72.12	−0.21	298	−8.83
			318	−7.44
			338	−0.20
βCD-HDI	−51.90	−0.18	298	0.19
			318	4.37
			338	7.15

The negative value of entropy (Δ*S*°) obtained for both polymers could be due to the decrease in randomness at the solid/solution interface [45]. Basically, the change of Δ*S*° value is related to the displacement of the adsorbed water molecules by the adsorbate [46]. Thus, the negative Δ*S*° value obtained in this study suggests that there is a reduction in randomness of adsorbate–solution interface during the adsorption process and displacement of water molecules by 2,4-DNP molecules on both polymers.

The calculated Gibbs free energy change, or Δ*G*° value, for βCD-HDI, is 0.19, 4.37 and 7.15 kJ mol^−1^ at 298, 318 and 338 K, respectively. The Δ*G*° value increased as the temperature elevated, which indicates that there are sufficient driving forces for the feasibility of adsorption at higher temperature. However, negative Δ*G*° values of βCD-TDI at the studied temperatures reveals that there is a decrease in the feasibility of adsorption at higher temperature, and yet it is thermodynamically feasible, spontaneous and chemically controlled [47].

### 3.6. Regeneration of the polymers

**Figure 17.**
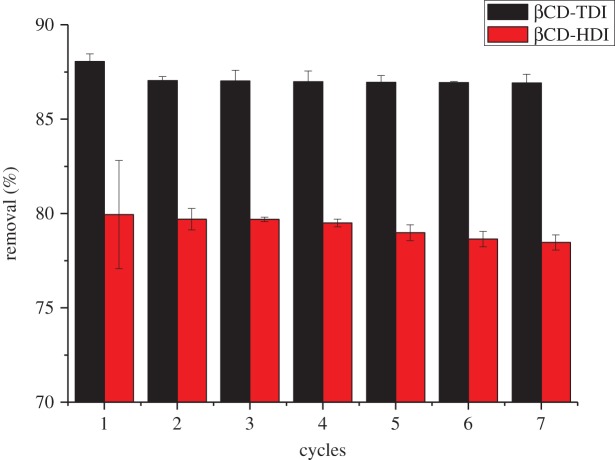
Recycling efficiency of both polymers on the removal of 2,4-DNP.

For the regeneration in this work, both used polymers were constantly stirred with acetonitrile and dried in the oven for 1–2 h. Adsorbent regeneration ability is vital to the practical application, as it will reduce the overall cost for the adsorbent. The reusability of both adsorbents was investigated as shown in figure 17. The experiment was repeated for seven cycles using the same βCD polymer. No significant degradation was observed in the sorbent performance and the removal efficiency was still well above 80% for βCD-TDI and 70% for βCD-HDI. These results showed that the adsorbents can be recycled for 2,4-DNP adsorption, and demonstrated that both polymers are stable and there is no carryover of the targeted analytes occurring during the removal process.

Comparatively, βCD-TDI showed a better removal efficiency than βCD-HDI, indicating its ability as a good adsorbent to remove 2,4-DNP from aqueous solutions. The reusability process using the synthesized βCD polymer is very effective due to the sorption mechanism, which was probably simultaneously dominated by van der Waals force, inclusion complex, π–π interaction and hydrogen bonding.

### 3.7. Comparison of the polymers with other reported methods

To demonstrate the potential use of βCD-TDI and βCD-HDI as sorbents in the removal study, the present method was compared with several published methods using different sorbents to remove 2,4-DNP compound from water samples. The experimental results for the different sorbents are summarized in table 7. It is observed from the table that the adsorption capacity and other parameters of the proposed polymers are better or comparable to the other reported adsorbents. Therefore, it can be concluded that βCD polymers have potential to be used as alternative sorbents for the removal of phenolic pollutants in a water body.

**Table 7.** Comparison with other reported sorbents for removal of 2,4-DNP. MIP, molecular imprinted polymer; IL, ionic liquid.

adsorbate	sorbent	sorbent dose (g l^−1^)	*C*_o_ (mg l^−1^)	*q*_e_ (mg g^−1^)	*q*_t_ (h)	ref.
2,4-DNP	cellulose acetate-MIPs	1	10	3.290	1	[48]
	polystyrene-MIPs	1	10	3.090	2	[48]
	bentonite	—	5–30	3.92	—	[49]
	Ti-Si-IL	2	5–100	5.189	1/2	[16]
	βCD-TDI	2	10	3.895	2	this work
	βCD-HDI	2	10	3.438	2	this work

### 3.8. Analysis of real samples

In order to validate the real sample applicability and suitability of the proposed method, the method was applied to analyse four different types of environmental water samples (tap, river, lake and sea water). The results indicated that no residues were detected in these samples. To investigate the effect of sample matrices on the removal efficiency, the samples were spiked with a concentration of 10 mg l^−1^ of 2,4-DNP. Three replicates of each of the samples were performed under optimal conditions.

**Figure 18.**
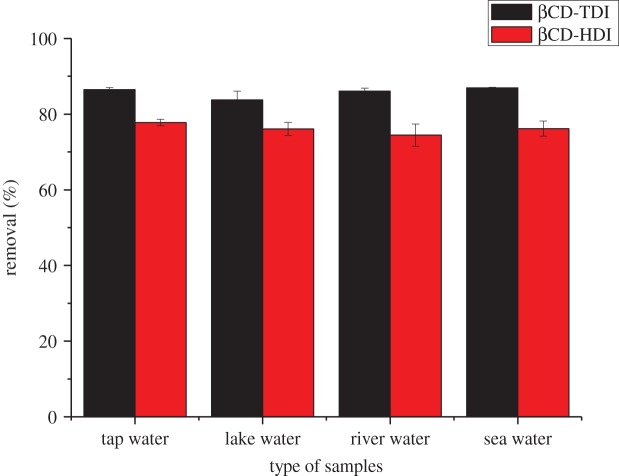
Removal efficiency of 2,4-DNP in various environmental water samples using βCD-TDI and βCD-HDI as adsorbent.

The corresponding results are presented in figure 18. As depicted, the βCD-TDI shows approximately greater than 86.0% 2,4-DNP removal while βCD-HDI shows greater than 76.0% of the removal. The obtained results indicated that the matrix of different samples has no significant effect on the removal efficiency of the target compounds. The removal efficiency is certainly acceptable, and the results demonstrated that both βCD polymers were very suitable for the removal of phenolic compounds in water samples without derivatization. Nevertheless, βCD-TDI showed better removal capability than βCD-HDI due to the structure effect of the bifunctional isocyanate linker used, as discussed.

## 4. Conclusion

In this study, mesoporous βCD polymers (βCD-TDI and βCD-HDI) were successfully synthesized and characterized, and their ability to remove 2,4-DNP in environmental water samples was compared. The overall reaction procedures were simple, cost-effective and easy to perform. Parameters affecting the removal efficacy were optimized and the effects of cross-linker in terms of the chemical structure and chemical properties were discussed. The batch study suggested that βCD-TDI shows better removal efficiency of 2,4-DNP than βCD-HDI due to the effects of the cross-linkers used. The aromatic TDI cross-linker exhibited higher reactivity than the aliphatic HDI cross-linker owing to a stronger π to π stacking interaction which does not exist in the βCD-HDI polymer, despite the host–guest or inclusion complex interaction, van der Waals forces and hydrogen bonding interaction. Furthermore, the aromatic TDI cross-linker is able to form a more rigid and thermally stable polymer than the aliphatic HDI cross-linker. Owing to the aromatic ring structure of 2,4-DNP, βCD-TDI polymer reacts in the same way, which is not observed with the HDI cross-linker. The small surface area of the βCD-TDI polymer also provides more active binding sites for the targeted analytes. In summary, a cross-link polymer provides insolubility, rigidity and stiffness to the polymer, which offers potential applications in various analytical applications. This study proposes that the aromatic TDI cross-link polymer performs better than the aliphatic HDI cross-linker polymer for the removal of aromatic ring based compounds, and can ascertain the role of the cross-linking network in the sorption process.

## Data accessibility

This article does not contain any additional data.
